# MOSAiC-ACA and AFLUX - Arctic airborne campaigns characterizing the exit area of MOSAiC

**DOI:** 10.1038/s41597-022-01900-7

**Published:** 2022-12-29

**Authors:** Mario Mech, André Ehrlich, Andreas Herber, Christof Lüpkes, Manfred Wendisch, Sebastian Becker, Yvonne Boose, Dmitry Chechin, Susanne Crewell, Régis Dupuy, Christophe Gourbeyre, Jörg Hartmann, Evelyn Jäkel, Olivier Jourdan, Leif-Leonard Kliesch, Marcus Klingebiel, Birte Solveig Kulla, Guillaume Mioche, Manuel Moser, Nils Risse, Elena Ruiz-Donoso, Michael Schäfer, Johannes Stapf, Christiane Voigt

**Affiliations:** 1 Institute for Geophysics and Meteorology, University of Cologne, Cologne, Germany; 2 Leipziger Institute for Meteorology, University of Leipzig, Leipzig, Germany; 3 Alfred-Wegener-Institut, Helmholtz-Zentrum für Polar- und Meeresforschung, Bremerhaven, Germany; 4 BreezoMeter, Haifa, Israel; 5 A.M. Obukhov Institute of Atmospheric Physics of the Russian Academy of Sciences, Moscow, Russia; 6 Laboratoire de Météorologie Physique, Université Clermont Auvergne/OPGC/CNRS, UMR 6016 Clermont-Ferrand, France; 7 Institute for Physics of the Atmosphere, Deutsches Zentrum für Luft- und Raumfahrt, Wessling, Germany; 8 Institute for Physics of the Atmosphere, Johannes Gutenberg University, Mainz, Germany

**Keywords:** Atmospheric science, Physics

## Abstract

Two airborne field campaigns focusing on observations of Arctic mixed-phase clouds and boundary layer processes and their role with respect to Arctic amplification have been carried out in spring 2019 and late summer 2020 over the Fram Strait northwest of Svalbard. The latter campaign was closely connected to the Multidisciplinary drifting Observatory for the Study of Arctic Climate (MOSAiC) expedition. Comprehensive datasets of the cloudy Arctic atmosphere have been collected by operating remote sensing instruments, in-situ probes, instruments for the measurement of turbulent fluxes of energy and momentum, and dropsondes on board the AWI research aircraft Polar 5. In total, 24 flights with 111 flight hours have been performed over open ocean, the marginal sea ice zone, and sea ice. The datasets follow documented methods and quality assurance and are suited for studies on Arctic mixed-phase clouds and their transformation processes, for studies with a focus on Arctic boundary layer processes, and for satellite validation applications. All datasets are freely available via the world data center PANGAEA.

## Abstract (summary)

**Table:** 

Measurement(s)	navigation data • temperature of air • atmospheric humidity • radar reflectivity • brightness temperature • cloud properties • radiation • atmospheric wind
Technology Type(s)	GPS navigation system • dropsondes • radar • microwave radiometer • particle count and size analyzer • imager • noseboom sensors
Factor Type(s)	temporal • location
Sample Characteristic - Environment	atmosphere
Sample Characteristic - Location	Arctic Ocean region

## Background & Summary

During the last decade, an unprecedented change of climate has been observed especially in the Arctic regions and is seen in many climate variables. Most obvious is the strong decrease in sea ice extent and thickness^1–3^, precipitation is observed more frequently as rain^4^, and the lower tropospheric temperature is rising much faster in the Arctic than in all other regions of the world^5^, a phenomenon called the Arctic amplification^6^. Key processes for the enhanced warming have been investigated^7–12^ showing a clear need to better understand the governing feedback mechanisms related to changes in surface albedo, water vapor, clouds, and lapse rate. Together with these local processes, also the role of meridional transport into and out of the Arctic needs to be investigated in more detail.

The German DFG project - TRR 172, “ArctiC Amplification: Climate Relevant Atmospheric and SurfaCe Processes, and Feedback Mechanisms (AC)^3^”^13,14^, a joint research initiative of the Universities Leipzig, Cologne, and Bremen and of the research institutes TROPOS (Leipzig) and Alfred Wegener Institute (AWI Bremerhaven and Potsdam), is investigating the processes and feedback mechanisms related to Arctic amplification by model studies and observations. To bridge the gap between localized ground based observations with a high temporal resolution and satellite borne observations providing a good areal coverage, but poor resolution in time and space, airborne measurements are well suited to study atmospheric processes especially close to the sea ice edge, where surface conditions change on small scales. Therefore, several airborne campaigns over the Arctic ocean have been conducted as part of (AC)^3^ with either one or both of the AWI polar research aircraft Polar 5 and 6^15^. The focus of these campaigns was on the observation of Arctic mixed-phase clouds and of the polar boundary layer in different seasons: the Arctic CLoud Observations Using airborne measurements during polar Day (ACLOUD^16–18^) in late spring and early summer 2017 based in Svalbard and the Polar Airborne Measurements and Arctic Regional Climate Model Simulation Project (PAMARCMiP) in spring 2018 out of Villum research station (Greenland). In this study we introduce and describe two follow-up campaigns that aim to extend the dataset, namely the Airborne measurements of radiative and turbulent FLUXes of energy and momentum in the Arctic boundary layer (AFLUX^19^) in early spring 2019 and the MOSAiC Airborne observations in the Central Arctic (MOSAiC-ACA^20,21^) campaign in late summer 2020 which was the airborne component of the Multidisciplinary drifting Observatory for the Study of Arctic Climate (MOSAiC^22^) project.

Given the remoteness and difficult logistics, only very few measurement sites provide detailed and continuous insights into the Arctic climate system. The Ny-Ålesund Research Station in Svalbard is one of the few examples, with e.g., the atmospheric observations at the German-French AWIPEV research base that is operated jointly by the Alfred Wegener Institute Helmholtz Centre for Polar and Marine Research (AWI) and the French Polar Institute Paul Emile Victor (IPEV). However, for a full understanding, detailed information on the atmospheric state and its interaction with the surface is needed across the full Arctic, which can not be provided by ground based observations at a fixed location. The complex transition between open ocean and sea ice with the highly heterogeneous marginal ice zone is also challenging for the interpretation of satellite measurements. Therefore, airborne measurements can fill an important gap to sense boundary layer processes and cloud development in this critical region. Thus, the general goal of the AFLUX and MOSAiC-ACA campaigns was to obtain a comprehensive dataset of atmospheric parameters in the polar cloud-covered and cloud-free atmospheric boundary layer (ABL) and lower troposphere over compact sea ice, the marginal sea ice zone, and open ocean. Research flights were planned in conjunction with atmospheric modeling such that they targeted specific conditions as, e.g., cold air outbreaks. Specific flight patterns aimed to assess radiative and turbulent fluxes, thermodynamic profiles, and cloud macro- and microphysical properties. The combined analysis of the measurement data and modeling efforts set up for the observed cases can be used to estimate the role of Arctic clouds and surface heterogeneities for the amplified climate change in polar regions. Furthermore, to get a grasp on the seasonal variability, a comparison of the observations from all campaigns that were carried out as part of (AC)^3^ during episodes of several weeks in different seasons, is highly valuable. With the comprehensive dataset collected with the two campaigns, it is possible to perform various process studies and to evaluate satellite observations and atmospheric model simulations. For example, the data provide information on the life cycle of Arctic mixed-phase clouds, the partioniong into liquid and ice, the composition and amount of precipitation, or the impact of mixed-phase clouds on the radiation budget can be studied by these data. Furthermore, the dataset can be used to develop and improve model parameterizations and serve as critical test data to assess the performance of atmospheric models including the application of appropriate forward operators on the model output to mimic the measured quantities. Moreover, due to collocation with satellite overpasses the dataset and derived parameters can be used for validation purposes or the development of retrieval algorithms of cloud and surface parameters. By the *ac3airborne*^23^ python module, the collected datasets will be more visible and very easy usable by other interested scientists in future studies.

## Methods

This section provides an overview of the platform operated during the campaigns, the campaigns itself, and the design of the research flights, followed by a more in-depth description of the aircraft scientific payload. For each instrument the corresponding data acquisition, the processing steps performed to create the published final datasets, and the data contained in the published datasets are described.

### Platform and campaign set up

The data presented are based on measurements conducted with the AWI research aircraft Polar 5^15^, a former Douglas DC-3 specifically modified by Basler Turbo Conversions for flying under extreme polar conditions. In the following, it is referred to as Basler Turbo-67 (BT-67). Together with its sister aircraft Polar 6, it belongs to AWI and is operated by Kenn Borek Air Ltd. Canada. The aircraft is unpressurized, has an endurance of 5 to 6 h, and is able to fly at low levels down to 200 ft and at low speed (60 m s^−1^) for in-situ measurements, e.g., of meteorological parameters.

**Fig. 1:**
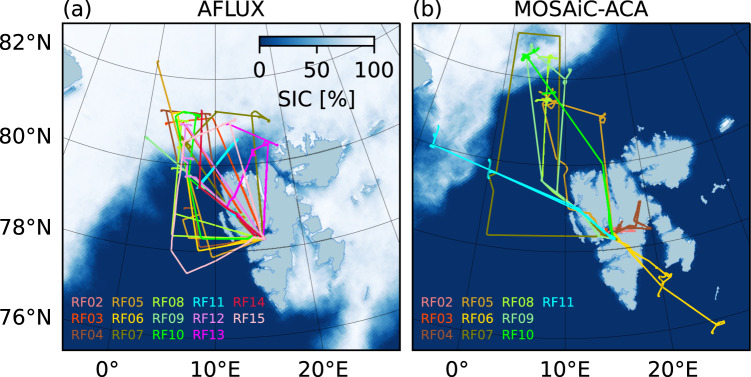
Tracks of the research flights performed northwest of Svalbard during AFLUX (**a**), and MOSAiC-ACA (**b**). Background shows the sea ice concentration averaged over the respective campaign period (19 March to 11 April 2019 for AFLUX and 30 August to 13 September 2020 for MOSAiC-ACA) as derived by University of Bremen from AMSR-2 measurements^120^.

During the two campaigns, Polar 5 was based in Longyearbyen (N 78°13′, E 15° 38′, Svalbard, Norway) and most flights were performed northwest of Svalbard over the Fram strait covering both sea ice free ocean and the marginal sea ice zone. Thereby, AFLUX took place in spring 2019 (19 March - 11 April) and MOSAiC-ACA in late summer 2020 (30 August - 13 September) during the MOSAiC drift experiment. Details on the flights (dates, take-off, landing, flight hours) are summarized in Table 1. The corresponding flight tracks are given in Fig. 1a,b. All flights were performed during day light hours, with a typical flight duration between 4 and 6 hours. During AFLUX, 14 research flights with in total 67 flight hours have been performed, whereas during MOSAiC-ACA ten flights with 44 hours, respectively.

**Table 1:** List of research flights (RF) conducted out of Longyearbyen (Svalbard) during AFLUX and MOSAiC-ACA.

#RF	Date	Take-off & Landing	Duration	Scientific Target
**AFLUX**				
RF02	2019-03-19	16:35-17:55	1:19 h	Test of instrumentation.
RF03	2019-03-21	09:51-14:31	4:39 h	Cloud structures and impact on fluxes over sea ice
RF04	2019-03-23	11:27-16:55	5:28 h	Sampling of cloud microphysics and fluxes in the boundary layer and/or in the low-level clouds over sea ice; Studying of cold air-mass and boundary layer evolution and cloud structure over open water
RF05	2019-03-24	10:01-14:51	4:49 h	Clouds during cold air outbreak; Turbulent and radiative energy fluxes over different surfaces
RF06	2019-03-25	10:37-15:50	5:13 h	Remote sensing and in-situ measurements in a cold-air outbreak over Fram Strait
RF07	2019-03-30	10:13-15:27	5:14 h	Turbulent and radiative energy fluxes and cloud microphysics in different cloud layer conditions
RF08	2019-03-31	08:58-14:28	5:29 h	Clouds in a strong cold air outbreak over sea ice close to the MIZ and open water
RF09	2019-04-01	07:35-12:37	5:20 h	Validation of satellite observations
RF10	2019-04-03	10:21-14:58	4:37 h	Turbulent, radiative flux measurements and microphysics in mid-level clouds and over sea ice
RF11	2019-04-04	08:38-12:26	3:47 h	Characterize clouds and surface fluxes ahead of a warm front over sea ice
RF12	2019-04-06	10:24-15:51	5:26 h	Vertical profiles of fluxes and cloud particles
RF13	2019-04-07	07:21-12:16	4:55 h	A-Train co-location with remote sensing and in-situ; Turbulent energy and momentum fluxes
RF14	2019-04-08	09:05-13:53	4:48 h	Characterize clouds and surface fluxes over sea ice
RF15	2019-04-11	09:37-15:14	5:37 h	Vertical profiles of fluxes and cloud particles
**MOSAiC-ACA**				
RF02	2020-08-30	08:14-09:07	0:53 h	Test of instrumentation
RF03	2020-08-31	10:20-10:58	0:38 h	Certification flight (PMS instruments)
RF04	2020-08-31	12:40-14:55	2:14 h	Joint P5 and P6 operation close to Longyearbyen; Test flight for P5 instruments
RF05	2020-09-02	06:55-12:23	5:27 h	A-train co-location north of Svalbard; Nose boom, radiation, and microwave radiometer calibration
RF06	2020-09-04	12:11-17:41	5:29 h	Atmospheric structure along the transition from a cloud-free region to a cloudy region during warm air intrusion
RF07	2020-09-07	08:22-14:05	5:42 h	Remote sensing of clouds in different regimes; Thermo-dynamic structure of the atmosphere and the wind field.
RF08	2020-09-08	08:00-14:05	6:40 h	Atmospheric structure over sea ice and open ocean
RF09	2020-09-10	08:30-14:45	6:14 h	Cloud evolution along wind direction over sea ice and open ocean; Evaluation of lee effects from Svalbard
RF10	2020-09-11	08:19-13:59	5:39 h	Lee effect of Svalbard on atmosphere and cloud conditions; Profile multi-layer clouds over sea ice and over open ocean
RF11	2020-09-13	09:20-15:06	5:46 h	Atmospheric structure over sea ice and open ocean

**Fig. 2:**
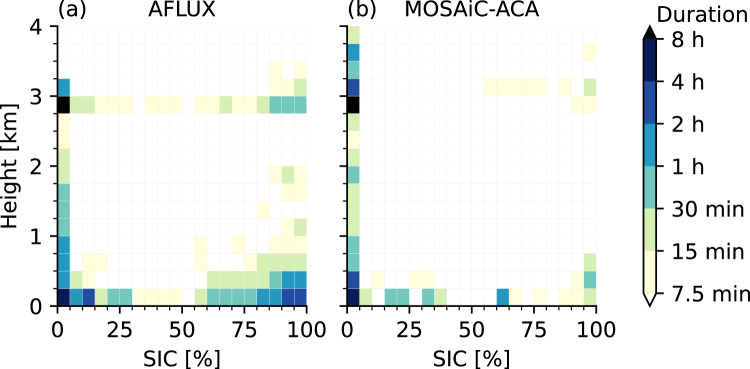
Flight time in hours as a function of the overflown sea ice concentration^120^ and flight altitude for all research flights during AFLUX (**a**) and MOSAiC-ACA (**b**) as shown in Fig. 1 excluding sections over land.

The two different seasons exhibited different environmental conditions with much less sea ice during MOSAiC-ACA (Fig. 1) and, as could be derived from dropsonde launches during the campaigns, warmer near-surface temperatures in the measurement region over sea ice and ocean (between −5 and +15 °C) in contrast to AFLUX (−27 and −2 °C). Correspondingly, the sea ice edge during MOSAiC-ACA was very far from Longyearbyen, at about 82°N north of Svalbard and 2° W west of Svalbard resulting only in very few flight hours over sea ice as shown in Fig. 2 by the distribution of flight hours according to flight altitude and different surface conditions. Low-pressure systems arriving at the Svalbard Archipelago sometimes lead to low cloud ceilings and precipitation, strong winds and heavy turbulence over Svalbard mountains that did not allow take-off at Longyearbyen. On several days heavy snowfall developed during AFLUX in Svalbard, whereas during MOSAiC-ACA many days with rain occurred. Flights were planned according to the weather situation aiming to assess the cloudy boundary layer over sea ice and ocean and in particular its development during cold air outbreaks. The scientific targets of each flight are listed in Table 1.

### Flight strategies

**Fig. 3:**
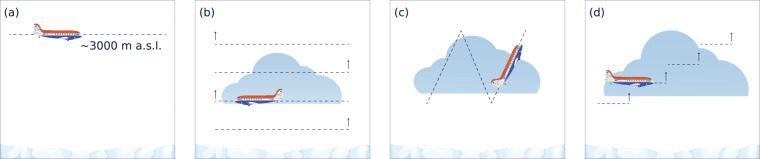
Different types of flight patterns flown during the campaigns: (**a**) remote sensing leg and (**b**) racetrack, (**c**) saw tooth, and (**d**) staircase pattern.

The flight strategies can be grouped into three different measurement approaches: (1) remote sensing of Arctic mixed-phase clouds and their transformation processes (e.g., glaciation, precipitation formation, cloud dissipation); (2) in-situ probing of Arctic mixed-phase clouds; (3) measurements of turbulent fluxes of sensible heat and momentum over the ocean and sea ice. The resulting flight patterns designed to achieve these targets are illustrated in Fig. 3 and are described in the following.

**Fig. 4:**
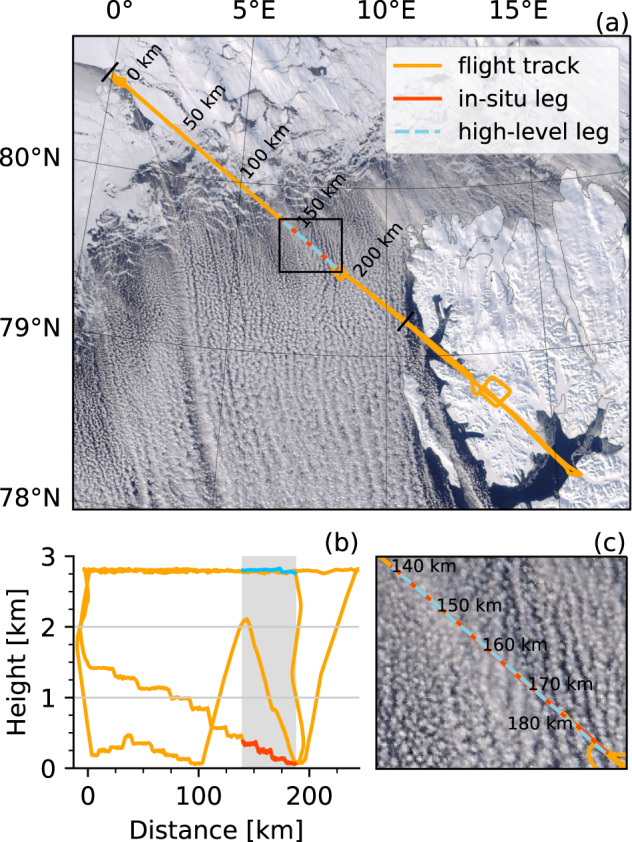
Flight track (orange) of RF09 from AFLUX on 1 April 2019 on top of an early afternoon Terra/MODIS composite from NASA worldview https://worldview.earthdata.nasa.gov. (**b**) shows the Polar 5 flight altitude as a function of along-track distance and a detailed view of cloud streets (**c**) near the in-situ (red) and high-level (blue) sections corresponding to Figs. 5 and 6.

1. Remote sensing measurements aimed to provide datasets for comparison with and as reference for satellite measurements, however, with much finer resolution. Flights were typically performed along straight legs over long distances at altitudes above 3000 m as needed for downward looking lidar measurements for eye safety reasons (Fig. 3a). The high flight altitude also ensured that observations are well above the top of typical low level clouds with a distance between aircraft and cloud top of at least 200 m, which is required to obtain an overlap of the sending and receiving antenna beam for radar and lidar.
  Legs were chosen to cover different surface types, i.e., open ocean, the marginal sea ice zone, and closed sea ice, either one type after another during one leg or during different legs on the same flight. Depending on weather conditions the legs were mostly chosen to be either along or across the mean atmospheric flow.
  Straight legs were included in almost all research flights on the transits to the target area often passing the AWIPEV station at Ny-Ålesund (N78° 55′, E11° 56′, Svalbard, Norway)^24^ for measurement comparisons. Occasionally, underflights of the A-Train satellite constellation^25^ have been included for same purposes. During such under-flights, a high-level leg of approx. 20 min duration along the satellite track has been combined with a successive in-situ pattern (see Fig. 4) along the same path in opposite direction. This pattern was either a staircase or sawtooth pattern or a combination of both. Typically, these high level legs have been supported by launching dropsondes to derive vertical profiles of the atmospheric state, i.e., pressure, temperature, humidity, and wind speed and direction.
2. In-situ probes measuring cloud and aerosol particles require exposure times long enough for sampling a sufficient air volume, which are different for the various probes. Depending on the actual situation either racetrack, sawtooth, or staircase pattern were chosen to measure cloud microphysical properties. For safety reasons, each pattern started with a descend from above cloud top to below cloud base to estimate vertical extent, structure, and characteristics of the cloud layer to refine the strategy and to avoid icing conditions.
  For racetracks (see Fig. 3b), horizontal legs along the same path, in alternating direction and stacked at different altitudes above, below, and within the clouds and precipitation have been performed. The duration of the legs was typically around 4 to 5 min. The vertical spacing of the legs depended on the vertical extent of the cloud layers and was adjusted in flight. The lowest possible flight level was 200 ft (60 m) above ground. Sawtooth patterns (Fig. 3c) were typically flown from below cloud base at 200 ft to above cloud top and vice versa with a typical climb or sink rate of 1000 ft min^−1^ (300 m min^−1^) along a horizontally straight line. For the staircase pattern (Fig. 3d), level legs in different altitudes are concatenated to each other. The vertical distribution of the legs is similar to the one for racetrack patterns, but they are not stacked above each other but flown along a straight line. This is usually done to sample clouds on a longer distance, assuming that the cloud structure does not significantly change and shows some kind of homogeneity of cloud properties over the long horizontal extent. Most of the time when in-situ patterns have been conducted, they were combined with one or multiple remote sensing legs over the same area to bring together those two measurement types.
3. Turbulent fluxes of sensible heat and momentum over different surface types were derived from measurements during three main types of flight patterns. First, long legs along a straight line were flown at about 200 ft (60–70 m) height to obtain near-surface fluxes. In convective conditions with a deep ABL this is sufficiently low for the detection of surface fluxes. In very stable conditions with a shallow ABL, this low level can already belong to the upper part of the ABL. In this case, the fluxes measured in this flight altitude can not be referred as surface fluxes. Second, vertical profiles of fluxes are derived from a series of several horizontal legs over each other in the same vertical plane. Thereby, if possible, the lowest leg has been flown also in an altitude of 200 ft. This pattern is similar to the before mentioned racetrack but with flight levels selected according to the vertical extent and structure of the ABL. The selection of the levels was done in flight based on profile measurements to determine the structure of the the ABL and especially its height. Therefore, measurements started with a descend from higher altitudes to the lowest possible level before the flux measurement patterns. Third, continuous vertical profiles of turbulent fluxes were obtained also from flights with low descend rates (200 ft/min).

Finally, at least once per campaign various instruments required specific maneuvers for their calibration, which have been included in the flights .

### Instrument description

Polar 5 has been equipped with very similar payload during AFLUX and MOSAiC-ACA, that can be grouped into the two major categories remote sensing and in-situ instruments. Within the remote sensing payload, active instruments like a cloud radar and lidar are operated together with passive instruments, namely microwave, spectral solar, and infrared radiometers, imaging spectrometers, fish-eye camera, and a sun photometer. The in-situ payload attached to the fuselage or the wings of Polar 5 can be used to characterize hydrometeors in a size range from 3 to 6400 μm. In addition to the remote sensing instrumentation and the in-situ probes, measurements providing the basic meteorological variables have been operated on Polar 5. For high-frequency measurements of wind vector, humidity, and air temperature in front of the aircraft a nose boom has been operated. The vertical profile of basic meteorological variables have been provided by a dropsonde system. All instruments are described in more detail below along with their configuration for the campaigns. Table 2 summarizes the instruments used in both campaigns on Polar 5 along with corresponding parameters measured. Collections of the datasets have been compiled and can be found on the public database PANGAEA for both campaigns, AFLUX^26^ and MOSAiC-ACA^27^. A description of the file formats and the structure of the datasets is given in each instrument subsection and in the Data Records section.

**Table 2:** Overview of the instrumentation on Polar 5 during AFLUX (A) and MOSAiC-ACA (M) and the measured quantities, where the number behind the campaign indicates the number of research flights (RFs) the instrument has been operational.

Instrument	Measured quantities, range, and sampling frequency	Campaign (#RFs)
**Meteorology**		
Dropsondes (RS904)^106^	Profiles of *T*, *p*, RH, Horizontal Wind Vector, 1 Hz	A(9),M(7)
**Turbulence**		
Nose boom^28,115^	*T*, *q*, *p*, Wind Vector, 100 Hz	A(13),M(10)
**Radiation**		
CMP-22 Pyranometer^62,63^	Solar Irradiance (Upward, Downward, Broadband *λ* = 0.2–3.6 μm), 20 Hz	A(14),M(10)
CGR-4 Pyrgeometer^62,63^	Terrestrial Irradiance (Up- and Downward, Broadband *λ* = 4.5–42.0 μm), 20 Hz	A(14),M(10)
SMART -Albedometer^52^	Spectral Irradiance (Up- and Downward *λ* = 0.4–1.8 μm), 2 Hz Spectral Radiance (Upward, FOV = 2.1°, *λ* = 0.4–1.0 μm), 2 Hz	A*, M(8)
**Remote Sensing**		
AISA Eagle/Hawk^116^	Spectral Radiance (Upward, Swath = 36°, *λ* = 0.4–2.5 μm), 20–30 Hz	A(13),M(7/5)
Fish-Eye/Wide-Angle Camera^117^	Spectral Radiance (Lower Hemisphere, RGB Channels), 4–6 s	A(13),M(8)
AMALi^46^	Particle Backscattering Coefficient (*λ* = 355,532 nm), Cloud Top Height, Particle Depolarization (*λ* = 532 nm), 1 s	A(12),M(5)
MiRAC-A^35,118^	Radar Reflectivity Factor, Doppler Spectra, *ν* = 94 GHz, tilted by 25°, 1 s Brightness Temperature (BT), *ν* = 89 GHz, tilted by 25°, 1 s	A(14),M(7)
MiRAC-P^35^	Brightness Temperature (BT), *ν* = 6 × 183.31,243,340 GHz, nadir, 1 s	A(14)
HATPRO^41^	Brightness Temperature (BT), *ν* = 7 × 22.24–31.4,7 × 51.26–58.00 GHz, nadir view, 1–2 s	M(9)
KT-19	Brightness Temperature (Upward nadir, *λ* = 9.6–11.5 μm), 20 Hz	A(14),M(10)
Sun Photometer^119^	Spectral Aerosol Optical Depth (AOD) *λ* = 400–2000 nm), 15 s	A(11), M**
**Cloud Microphysics**		
2D-S^90^	Cloud PNSD, Particle Shape, *D*_p_ = 10–1280 μm, 1 Hz	A(14),M(7)
Polar Nephelometer^82^	Cloud Prticle Scattering Phase Function, 1 Hz	A(14),M(7)
CAS^72^	Cloud PNSD, *D*_p_ = 3–50 μm, 1 Hz	A(13)
CDP^77^	Cloud PNSD, *D*_p_ = 3–50 μm, 1 Hz	M(8)
CIP^72^	Cloud PNSD, Particle Shape, *D*_p_ = 15–960 μm, 1 Hz	A(13),M(8)
PIP^72^	Precipitation PNSD, Particle Shape, *D*_p_ = 100–6400 μm, 1 Hz	A(13),M(8)
Nevzorov Probe^101^	LWC, TWC, 1 Hz	M(8)

*λ* is wavelength, *ν* is frequency, *T* is temperature, and *p* is atmospheric pressure. RH is relative humidity, FOV is field of view, PNSD is the particle number size distribution, and *D*_p_ symbolize the particle diameter. The references listed for each instrument describe the instrument, calibration procedures, or methods applied to ensure data quality. *Note, SMART only measured the spectral downward irradiance during AFLUX while upward was measured in both campaigns. **Note, data not yet processed.

#### Nose boom and navigation system

The basic sensor of the nose boom installed on Polar 5, are an Aventech (Aventech Research Inc., Canada) five-hole probe for high frequency pressure measurements, from which the wind vector can be derived that is placed at the tip of the nose boom and an open-wire Pt100 installed sidewards in a Rosemount housing. To avoid icing problems, the five-hole probe is equipped with a deicing system (designed by Aventech Research Inc., Canada) that ejects water during short flight sections not needed for the data analysis. Differential pressure transducers are of type Setra 239 R (Setra Systems Inc., MA, USA) for angle of attack, angle of sideslip, and for the dynamic pressure, while a Setra 278 provides the static pressure. The data are recorded at 100 Hz. A combination of a high-precision global positioning system (GPS) receiver and an inertial navigation system (INS) installed into Polar 5 is used to derive the wind vector in an earth-fixed coordinate system. The INS provides longitude, latitude, ground speed, and angular rates, which are necessary for the derivation of pitch, roll, and true heading angles. The accuracy is 0.1° for roll and pitch and 0.4° for true heading. The INS and GPS data were merged by complementary filtering.

The calculation of the wind vector follows a procedure based on an accurate calibration of the initial wind measurements using a combination of the differential measurement capabilities of the GPS and the high-accuracy INS^28^. Altogether, this finally results in horizontal wind components with an absolute accuracy of 0.2 m s^−1^ for straight and level flight sections^16^. We stress that vertical wind can only be analyzed as the deviation from the average vertical wind. For sections of several kilometers length, we obtain an accuracy of the vertical wind speed relative to the average wind of about 0.05 m s^−1^.

After correcting the temperature measurements for the adiabatic heating effect of the air by the dynamic pressure, an absolute accuracy of 0.3 K with a resolution of 0.05 K is reached.

The Polar 5 nose boom carried also a closed-path LI-7200 gas analyzer for CO_2_ and H_2_O concentration measurements^29^. For slow humidity measurements (frequency of 1 Hz), a Vaisala HMT-333 with a temperature and HUMICAP humidity sensor was mounted in a Rosemount housing. Based on the temperature measurements (uncertainty of 0.1 K), the humidity data were corrected for adiabatic heating and reach an accuracy of 2%^30^.

All data were recorded with a frequency of 100 Hz. However, it should be kept in mind, that the calibration of the 100 Hz data is only valid for straight and level flights, when using these for the calculation of turbulent fluxes. Turbulent fluxes are not provided in the dataset. This is left for the data user and can follow the examples given in^18,31,32^. We stress that only such flight sections should be considered for the derivation of turbulent fluxes where the aircraft is flying in a straight line. Note also that most flights during MOSAiC-ACA and those during AFLUX over sea ice were carried out in conditions with absolute values of heat fluxes below 20 W m^−2^. Such conditions with low fluxes represent a challenge for the accuracy as compared to conditions with strong convection and strong signals^16^. Nevertheless, the comparison of flights with Polar 5 and Polar 6 during ACLOUD using the same nose boom equipment has shown a remarkable agreement of both measurement systems^16,18^.

The processed datasets for the nose boom measurements are available in ascii format on PANGAEA in a 100 Hz resolution for thirteen flights of AFLUX^33^ and ten of MOSAiC-ACA^34^.

#### Radar

The Microwave Radar/radiometer for Arctic Clouds (MiRAC; designed by Radiometer Physics GmbH, Germany)^35^ has been designed for operation on board the polar research aircraft Polar 5 and 6. The active radar component (MiRAC-A) has been operated on Polar 5 on both campaigns AFLUX and MOSAiC-ACA. It consists of a single vertically polarized Frequency Modulated Continuous Wave (FMCW) cloud radar (RPG-FMCW-94-SP) at around 94 GHz and an additional horizontally polarized passive channel at 89 GHz using the same receiver for measuring the brightness temperature, that is used for the derivation of the liquid water path (LWP). MiRAC-A is operated in a bellypod fixed below the aircraft fuselage. To avoid saturation of the receiver due to strong ground reflection^36^, the radar is mounted pointing 25° backwards off nadir when assuming a leveled aircraft. The cloud radar provides vertically resolved profiles of the equivalent radar reflectivity as well as higher moments of the Doppler spectrum. The Doppler spectra and higher moments are not provided for airborne operation. The Doppler spectra and higher moments are not provided as airborne operation induces strong Doppler and aliasing effects^35^. For correction knowledge of the background wind field with a high resolution and areal coverage would be required, which is not available from measurements on Polar 5.

The vertical resolution of the raw data is given by the settings in the chirp sequences of the measurement program and is 4.5 m close to the aircraft (up to 500 m distance) and 13.5 m for the rest of the profile along the slanted path^35^. The processed final datasets^37,38^ have a constant vertical resolution of 5 m with respect to nadir view underneath the aircraft. To achieve this, a multi-step post-processing is applied^35^ that includes corrections and conversions of the signal: subtraction of mirror signal due to surface reflections, application of a speckle filter, correction for sensor altitude, mounting position, and pitch and roll angle^39^, and remapping onto the constant vertical grid of 5 m by taking into account each latitude and longitude position of each range bin. The temporal resolution is approximately 1 s which is the sum of the duration for both chirp sequences. Due to disturbances by surface reflections, the resulting regularly gridded data is only reliable from 150 m above ground level up to altitude of the aircraft.

The 89 GHz channel is especially sensitive to the surface emission and the emission by liquid clouds. Over the open ocean, where the emissivity of the surface is low, this channel can be used to retrieve the LWP^40^. Note, that the passive observations are for a slanted path of 25° and that no correction for the attitude and viewing geometry has been applied. As for the active measurements, the time resolution of the brightness temperature datasets is approximately 1 s.

The MiRAC-A datasets are available in netcdf format on PANGAEA for 14 flights of AFLUX^37^ and seven flights of MOSAiC-ACA^38^ in 1 s resolution. For both data, reflectivities and brightness temperatures, a flag indicating the instrument status is provided.

#### Microwave radiometers

During AFLUX and MOSAiC-ACA, passive microwave radiometers have been operated on board Polar 5 in addition to the passive channel at 89 GHz of the MiRAC-A radar. The radiometers have been mounted inside the cabin pointing nadir with respect to the aircraft fuselage. In the AFLUX configuration, the MiRAC-P (Radiometer Physics GmbH, Germany)^35^ radiometer has been operated with its six vertically polarized double sideband channels centered around the strong water vapor absorption line at 183.31 GHz and two horizontally polarized window channels at 243 and 340 GHz. The channels around the 183.31 GHz water vapor absorption line can be used to sense atmospheric moisture. The more the channels are displaced from the absorption line center, the lower in the atmosphere the emitted radiation originates, i.e., the lower the peak of the humidity weighting function and therefore the maximum of information is. Information on humidity from different layers can be derived by combining all spectral channels. With increasing frequency (243 and 340 GHz), larger snow particles can lead to a brightness temperature depression due to scattering effects so that these channels can give information on snow and ice water content. During MOSAiC-ACA, the radiometer operated was the Humidity And Temperature PROfiler (HATPRO; Radiometer Physics GmbH, Germany)^41^. It has seven vertically polarized channels along the water vapor absorption line at 22.24 GHz (K-band) and seven horizontally polarized ones close to the oxygen absorption complex at around 60 GHz (V-band). By the same principal as for MiRAC-P, the humidity channels can be used to retrieve humidity profiles and the ones in the oxygen complex could provide information on the temperature profile. In addition, by using channels in the K-band it is possible to derive the integrated water vapor (IWV) and LWP below the aircraft by appropriate retrieval algorithms^42,43^. In addition to atmospheric parameters, the passive microwave radiometers can be used to derive ocean surface as well as sea ice emissivities.

For MiRAC-P, data are available for all 14 flights of AFLUX, whereas HATPRO measured during nine flights of MOSAiC-ACA. The final datasets^44,45^ for both instruments have been corrected for non-physical brightness temperatures by hand and doubled time stamps have been removed. The uploaded datasets have a 1 s resolution and are available in netcdf format.

#### AMALi

The Airborne Mobile Aerosol Lidar (AMALi; AWI Potsdam, Germany) system^46^ has been operated onboard Polar 5 in both campaigns installed inside the cabin pointing nadir through the floor, thus, probing the atmosphere between the flight level and the surface. It is a backscatter lidar having three channels: one unpolarized channel in the ultraviolet at 355 nm and two channels in the visible spectral range at 532 nm (perpendicular and parallel polarized). For eye safety reasons, AMALi was operated at flight altitudes above 9000 ft only. Overlap between the transmitted laser beam and the receiving telescope is achieved for ranges larger than 235 m^46^. Data are recorded with 7.5 m vertical and 1 s temporal resolution. For consistency to the radar profiles, the AMALi data were converted into “altitude above sea level” by using the GPS altitude. To improve the signal-to-noise ratio, the profiles were averaged for 5 s temporal resolution, which yields a horizontal resolution of approximately 350 m for typical aircraft speed during measurements.

The backscattered intensities can be converted into attenuated backscatter coefficients, depolarization ratio at 532 nm, and the color ratio (532 to 355 nm) to analyze cloud and aerosol particles (not provided in the dataset). The data processing eliminated the background signal, which mainly results from scattered sunlight and electronic noise. Additionally, a drift of the so-called baseline of each channel was corrected for. Neglecting aerosol extinction, the attenuated backscatter coefficients for each channel were calculated from the background-corrected signals by normalizing the measurements to a typical air density profile^47^. This is done by using data from the AWIPEV^24^ station in Ny-Ålesund.

The published dataset provides cloud top heights derived from the lidar profiles in 1 s resolution and by that as well the cloud mask. Clouds below the aircraft were identified from the attenuated backscatter coefficients in the 532 nm parallel channel. Each height bin of the profile, which exceeds the backscatter coefficients of a reference cloud-free section of five bins above the possible cloud detection by a factor of five, was labeled as a cloud. Cloud top height was then defined as the highest altitude, which meets the above criterion for consecutive altitude bins.

In the published datasets, cloud tops in close distance to the aircraft (less than 100 m below the flight level) and low clouds (below 30 m above the ground) are excluded. The datasets are available in netcdf format and 1 s resolution on PANGAEA for twelve flights of AFLUX^48^ and five of MOSAiC-ACA^49^.

#### SMART

The Spectral Modular Airborne Radiation measurement sysTem (SMART; Leibniz-Institute for Tropospheric Research, Germany and Enviscope GmbH, Germany) is configured to measure the spectral solar irradiance and radiance. It is equipped with four optical inlets mounted at the fuselage of the Polar 5 and connected via optical fibers to grating spectrometers. These spectrometers disperse the incident radiation on a single-line photodiode array. Dark measurements are conducted with optical shutters. The upward-looking optical inlets are actively horizontally stabilized with respect to aircraft movement within pitch and roll angles of 5°^50^. Irradiance measurements by SMART cover a spectral range between 300 and 2200 nm, while spectral radiance is measured between 300 and 1000 nm only^16,51^. Both are sampled with a frequency of 2 Hz. Due to an increase of noise at the edges of the measured spectra, the final data are provided for 400 and 1800 nm wavelength, only. The measurement uncertainties are related to the radiometric and spectral calibration and to the correction of the cosine response which sum up to a total wavelength-dependent uncertainty ranging between 3 and 14%^52^.

During AFLUX, only the upward facing optical inlets for the observation of the downward radiance and irradiance could be installed. However, for most of the time, the measured signal was either contaminated by condensation on the inside of the optical inlets or the stabilization platform did not work properly. Therefore, the SMART dataset providing quality checked, radiometrically calibrated, and cosine corrected solar spectra (400–1800 nm) along the flight tracks, is only available in netcdf format with a 2 Hz resolution on PANGAEA for eight flights of MOSAiC-ACA^53^.

#### Spectral imager

The Airborne Imaging Spectrometer for Applications (AISA) Hawk^16,54^ and AISA Eagle^16,55^ (designed by Specim Ltd., Finland) were operated onboard the Polar 5 during AFLUX and MOSAiC-ACA. AISA Hawk consists of a downward-viewing push-broom sensor aligned across the flight track to measure 2-dimensional (2D) fields of upward radiance. It contains 384 across-track pixels, where each pixel delivers a whole spectrum in a wavelength range between 930 and 2500 nm in 288 channels with an average spectral resolution of 5.6 nm.

AISA Eagle is the second imager and uses a similar measurement technique like AISA Hawk, but covers a shorter wavelength range with a higher spectral and spatial resolution. In comparison to AISA Hawk, it has 1024 across-track pixel and 504 spectral channels to cover a wavelength range between 400 and 970 nm with 1.2 nm spectral resolution.

AISA Hawk and AISA Eagle have a field of view of 36° and used a sampling frequency of 20 Hz during both campaigns. However, the data were not recorded continuously throughout the whole flight. Measurement sequences of approximately 10 min duration were performed, whenever the conditions were appropriate (no in-cloud measurements, no measurements in too close distance to the cloud top or surface).

The quality checked and radiometrically calibrated datasets contain 2D fields of cloud top and surface spectral radiance observed along the flight track and are available on PANGAEA in netcdf format with 20 Hz resolution for thirteen flights of AFLUX^56^ and seven flights for AISA Eagle during MOSAiC-ACA^57^. Due to condensation on the quartz window of AISA Hawk in cold environments (high flight altitude), these data are only available for five flights during MOSAiC-ACA.

#### Nikon

To measure the directional distribution of upward radiance in the full lower hemisphere, a commercial digital camera (Nikon D5; Nikon Inc., Japan) was mounted at the bottom of the fuselage. The camera was equipped with a fish-eye lens during the campaigns, with the exception of the first half of MOSAiC-ACA, where a wide-angle lens was used. The camera recorded images every 4 to 6 s using three spectral channels (RGB) and allows cloud top and surface observations within a field of view of 80 × 100° (wide-angle lens, across × along track) and about 150° (fish-eye lens). All images were recorded in a raw data format to gain the full dynamic depth of the sensor (14 bit) and the full spatial resolution (5584 × 3728 pixels). The camera was calibrated with respect to its spectral, radiometric, and geometric characteristics for all camera settings (ISO value, shutter speed, and aperture) used during the flights.

Rectified angular-resolved fields (0.2° resolution) of calibrated radiances of the Arctic surface and cloud tops along the flight track for the three spectral bands (red, green, and blue) are provided. Combining the downward irradiance measured by SMART and the radiances from the fish-eye camera allows the calculation of the hemispherical-directional reflectance factor (HDRF) at flight altitude. Following the method described by^58^, the HDRFs of sea ice and open-ocean surfaces can be separated employing a sequence of surface images. Further, the Nikon data were used to classify the sea ice and ocean surface into open water, sea ice, and melt ponds based on color thresholds. Depending on the illumination conditions, these thresholds were determined using color intensity histograms which were created for training samples^59^.

The datasets are available on PANGAEA in netcdf format in a 4 to 6 s resolution for thirteen flights of AFLUX^60^ and eight of MOSAiC-ACA^61^, respectively.

#### Broadband radiation

Solar and terrestrial broadband irradiances were measured by a pair of upward- and downward-looking CMP22 pyranometers (spectral range of 0.2 to 3.6 μm) and CGR4 pyrgeometers (4.5 to 42 μm), respectively (both designed by Kipp & Zonen B. V., Netherlands). The sampling frequency of the radiometer is 20 Hz. Unlike SMART, the sensors are fixed to the aircraft frame. Therefore, the data processing includes a correction for the aircraft attitude and accounts for the sensor inertia.

In order to reconstruct fast changes of irradiance time series despite the slow sensor response, a deconvolution method was applied^62^. During AFLUX, time constants (*e*-folding time) of 1.4 s for the pyranometer and 3.6 s for the pyrgeometer were used as determined in the laboratory. For MOSAiC-ACA, the time constants were adjusted from in flight maneuvers of known irradiance changes (e.g., turns). The adjusted time constants amount to 1.8 s for the pyranometer and 3.4 s for the pyrgeometer. Remaining dynamic effects of the pyrgeometer may results from rapid changes of the ambient temperature, when the temperature of the silicon dome adapts faster than the sensor temperature^63^. Therefore, sections with a change of air temperature larger than 0.5 K min^−1^ were flagged, as indicated in the dataset. These data, which often refer to ascents and descents needs to be analyzed with care.

The downward solar irradiance was corrected for the aircraft attitude following a common geometric post-processing procedure^64^. This correction holds only for direct solar radiation and was applied only for manually identified sections that were dominated by direct illumination (e.g., cloud-free above the aircraft). In these conditions, the fraction of the direct downward solar radiation was be determined by radiative transfer simulations. The selection of cloud-free conditions might be uncertain. To allow user of the data to make their own decision, both uncorrected data (referring to cloudy conditions) and corrected data (referring to cloud-free conditions) are provided in the published solar downward irradiance dataset of MOSAiC-ACA^65^. Since the uncertainty of all broadband irradiances become large for roll and pitch angles of more than 5° these data were flagged and need to be analyzed with care.

The broadband irradiance measurements may also suffer during flights through super-cooled liquid clouds, when icing builds up at the radiometer domes. Sections which are likely to be influenced by icing were flagged for both the pyranometer and the pyrgeometer. In cloud-free conditions, pyranometer icing was identified by potential discrepancies between the measured and the simulated downward cloud free solar irradiance. Critical sections were checked using the observations of an on-board camera. However, especially during MOSAiC-ACA, the detection of icing was challenging and often unclear. Thus, large uncertainties remain for the pyranometers. The pyrgeometers seemed not to be affected by icing during MOSAiC-ACA.

The published datasets contain the upward and downward solar and terrestrial irradiances in netcdf format in 20 Hz resolution for all 14 flights of AFLUX^66^ and all ten flights of MOSAiC-ACA^67^ and are available on PANGAEA.

#### KT-19

The brightness temperature below Polar 5 was measured by an infrared radiation thermometer (KT-19.85II, short KT-19; Heitronics Infrarot Messtechnik GmbH, Germany) looking into nadir-direction. The instrument operates in a spectral range between 9.6 and 11.5 μm where the impact of atmospheric absorption is negligible. Thus, the brightness temperature measured in flight altitude is assumed to equal either the cloud top or the surface brightness temperature to a good approximation^68^.

The brightness temperatures of the KT-19 are measured at 20 Hz resolution and published in a joint dataset with the broadband irradiances measured by the pyranometers and the pyrgeometers and is available for the same flights, 14 AFLUX^66^ and eight MOSAiC-ACA^67^, respectively.

#### Sun photometer

The airborne Sun photometer with an active tracking system (SPTA; Dr. Schulz & Patner Buckow, Germany) was installed under a quartz dome of Polar 5 to derive the spectral aerosol optical depth (AOD). It operates a filter wheel with ten selected wavelengths in the spectral range from 367 to 1024 nm, one after the other for 1 s. To measure the direct solar irradiance, the optics of the SPTA use an aperture with a field of view of 1°. With knowledge of the extraterrestrial signal, the spectral optical depth of the atmosphere as well as spectral optical depth of aerosol was derived^69^. The extraterrestrial signal was calculated based on a Langley calibration, which are performed regularly in a high mountain area (Izana, Tenerife). The data were screened for contamination by clouds to minimize an artificial enhancement of the AOD. The cloud screening algorithm applied a threshold of measured irradiance and made use of the higher temporal and spatial variability of clouds compared to the rather smooth changes of aerosols properties^70^.

The final processed dataset has a resolution of 15 s and is available in ascii format for eleven flights of AFLUX^71^ on PANGAEA. For MOSAiC-ACA the data have not been processed yet.

#### Scattering cloud probes

Data recorded by the Cloud Aerosol Spectrometer (CAS; Droplet Measurement Technologies, Longmont, CO, USA) and the Cloud Droplet Probe (CDP; Droplet Measurement Technologies, Longmont, CO, USA) give the droplet size distribution from 3 to 50 μm^72–75^. Both instruments determine the particle drop size by the intensity of forward scattered laser light (4–12°, given by the manufacturer) underlying Mie theory. Standard methods for size calibration using mono-disperse glass beads have been applied^76^. For both instruments, the size of the sample area was determined by using a piezoelectric droplet generator setup similar to the design of Lance *et al*.^77^. For sizing the particles the Mie binning approach^78^ was applied with the refraction index of water (n = 1.333), including a distinct choice of bin edges to avoid ambiguities due to Mie resonances in the size range below 10 μm. Here the range of diameter can vary by a factor of two while data above 10 μm have reduced Mie oscillation and their uncertainty drops to ~30%^79^.

The final datasets include the total include the total particle number concentration, effective diameter, and liquid water content (LWC) in addition to the particle number concentration in each size bin. These datasets are published in netcdf format and 1 Hz resolution for thirteen flights of AFLUX^80^ (CAS) and eight flights of MOSAiC-ACA^81^.

The Polar Nephelometer (designed by Laboratoire de Météorologie Physique, France) measures the angular scattering coefficients (ASC, i.e., non-normalized scattering phase function in μm^−1^ Sr^−1^ Hz) of an ensemble of cloud particles (i.e., water droplets, ice crystals, or a mixture of both) from a few micrometers to approximately 1 mm in diameter^82^. The measurements are performed at a wavelength of 0.8 μm with scattering angles ranging from ±15 to ±162° and with an angular resolution of 3.5°. The average errors of measurements lie between 3 to 5% for scattering angles ranging from 15 to 162° (with a maximum error of 20% at 162°)^83^. Mean values of the calibrated non normalized scattering phase functions were computed each second and synchronized with the data recorded on the aircraft system. Electronic offsets of each channel were estimated based on the signal measured during clear air sequences. The background signal was then subtracted to the Polar Nephelometer cloudy signal^83^. ASC can be used to discriminate spherical from non-spherical cloud particles, as well as the dominant cloud thermodynamical phase^84,85^. In addition, the extinction coefficient and the asymmetry parameter g can be derived from these measurements^86,87^ with uncertainties of ~25% and ± 0.04, respectively.

The ASCs are provided with a temporal resolution of 1 Hz in netcdf format for 14 flights of AFLUX^88^ and seven of MOSAiC-ACA^89^ on PANGAEA.

#### Optical array probes

The basic measurement of optical array probes (OAP) is shadowgraphs of water and ice particles. Two-dimensional images of hydrometeors are reconstructed from individual slices, where a slice is the state (shadowed or non shadowed) of a linear multi element photo diode array at a given moment in time. The data recorded by the Cloud Imaging Probe (CIP; Droplet Measurement Technologies, Longmont, CO, USA), the Precipitation Imaging Probe (PIP; Droplet Measurement Technologies, Longmont, CO, USA)^72^, and the 2D Stereo Imaging Probe (2D-S; Stratton Park Engineering Company, Boulder, CO, USA)^90^ differ in pixel quantity and resolution (64 diode array with 15 μm resolution for CIP, 64 diode array with 103 μm resolution for PIP, and 128 diode array with 10 μm resolution for 2D-S). For observable particle size ranges, see Table 2. Before, after, and during the field campaigns, measurements with the spinning disk calibration tool from Droplet Measurement Technologies^91^ were done in order to check functionality and a consistent resolution of the CIP and PIP during the campaign period.

The sampling speed was set to a constant value corresponding to the highest achievable airspeed of 120 m s^−1^, to avoid loss of data due to a possible failure in live airspeed data. This provides an oversampling of the particle image. With validated true air speed data, raw images are squeezed to their correct frame afterwards. For data evaluation of the raw particle images recorded by the CIP and PIP, the processing software SODA (Software for OAP Data Analysis, provided by A. Bansemer, National Center for Atmospheric Research/University Corporation for Atmospheric Research UCAR, 2013) is used. Standard processing and correction options with SODA were applied: Circle-fit sizing method, shatter correction, stuck bit correction, pixel noise filter, stretch correction, and ‘center-in’ as the effective array width method. In addition, the sampling area is adjusted by applying dead time correction^92^ and with an adjusted depth of field constant z = 8.18, which has been identified by laboratory instrument characterization for the CIP and PIP. The ice water content (IWC) and LWC are retrieved using a mass-dimension relationship^93^. Note these data have to be handled to account for the respective cloud phase. The LWC is valid in pure liquid clouds and IWC in pure ice clouds. Additionally, total number concentration, effective diameter (ED) and median volume diameter (MVD) are provided. From the individual CIP and PIP datasets, a combined dataset including CAS/CDP was created, which contains a continuous size spectrum of hydrometeors from 3–6400 μm and microphysical properties including total number concentration, ED, MVD and an estimated cloud water content (CWC) derived from it. Here, for the CWC calculations particles smaller 50 μm are assumed as droplets and larger 50 μm as ice crystals, which is appropriate for Arctic mixed-phase clouds^94,95^.

Raw data recorded by the 2D-S were processed using code described in^96^. The particle number size distribution (PNSD) of hydrometeors are computed from the 2D-S images following the procedure similar as the one for the CIP and PIP during ACLOUD^16^. Two sets of PNSD are calculated for each probe channel (horizontal and vertical) using two different definitions of the cloud particle diameter: Circumpolar diameter (Dcc), the diameter of the circle encompassing the particle image (equivalent to circle-fit sizing in SODA) commonly used with the Brown and Francis^93^ (labeled with BF95) mass diameter relationship and equivalent diameter (Deq), the diameter of a circle which has the same surface as the particle image. It was previously shown that Deq is least subject to error in sizing due to out of focus deformation of the image^97^. Additionally, these PNSD are estimated when every particle images are considered, including truncated images, (suffix “ALL” or D0) and for complete images only (suffix “ALL-IN” or D1). Due to the large OAP measurement uncertainties for the smallest sizes, the first four size bins were removed and the PNSD is therefore documented for diameter ranging from 50 μm to a maximum of 2560 μm. Contamination of the measurements from shattering/splashing of ice/liquid particles on the instruments tips are also removed based on inter arrival time statistics and image processing^98^. Ice crystal size distributions are computed considering only non-spherical particle images which are identified based on their area (larger than 16 pixels) and circularity parameter (larger than 1.25)^99^. Total number concentrations and effective diameters of the sampled hydrometeors are derived from each PNSD whereas ice crystal effective diameter, ice median mass diameter, and IWC are calculated using the BF95 mass-diameter relationship.

The slight deviation in the PNSDs between the 2D-S (horizontal channel, using all images and Dcc as particle sizing) and the combined spectra of CAS, CIP, and PIP in Fig. 6a,b result from the different processing algorithms explained above^100^. Besides processing, matching measurements from different instruments with overlapping sizes is challenging because of their respective uncertainties^78^. However, the deviations here are within the scope of the uncertainties of the instruments.

The data of the OAPs are published on PANGAEA in netcdf format in 1 Hz resolution for 14 flights for 2D-S^88^ and thirteen for CIP and PIP^80^ of AFLUX, respectively. For MOSAiC-ACA, seven flights for 2D-S^89^ and eight for CIP and PIP^81^ are available. The combined dataset of CAS/CDP, CIP, and PIP is available with the same resolution and format have been published for thirteen AFLUX^80^ and seven MOSAiC-ACA^81^ flights, respectively.

#### Nevzorov

During the flight campaigns, a Nevzorov probe^101,102^ (designed by Sky Physics Technology, Woodbridge, ON, Canada) was installed on the fuselage of Polar 5 aircraft. The Nevzorov probe is a constant-temperature, hot-wire probe designed for the airborne bulk measurements of the LWC and total water content (TWC) of clouds in 1 Hz resolution. It has to be noted, that data recorded during a large temperature gradient, respectively during ascent and descent, might be inaccurate. Due to instrumental limitations ice fractions smaller 0.1 are difficult to resolve. As a result, it is challenging to retrieve both LWC and IWC in liquid dominated Arctic mixed-phase clouds^103^.

Due to an incorrect setting during AFLUX, only MOSAiC-ACA^81^ data for eight flights are published on PANGAEA in netcdf format and 1 Hz resolution.

#### Dropsondes

In total, 93 dropsondes were released from the Advanced Vertical Atmosphere Profiling System (AVAPS) installed on Polar 5 during both campaigns (33 during AFLUX, 60 during MOSAiC-ACA). The dropsondes measured vertical profiles of pressure, temperature, humidity, and the horizontal wind vector. The vertical resolution of the measurements was 5 to 6 m. With a sampling frequency of 2 Hz, this corresponds to a fall velocity of about 10 to 12 m s^−1^. The dropsonde type RD94 used during AFLUX was replaced by the new type RD41 during MOSAiC-ACA, which contains improved temperature and humidity sensors.

The Atmospheric Sounding Processing ENvironment (ASPEN, Version 3.4.4)^104^ software was used in two configurations to process the raw data. A quality check was performed with both configurations to remove invalid data points. The within the ASPEN software predefined configuration “research-dropsonde” further corrected for the response time of the temperature sensor. The inertia of the humidity sensor was not corrected for with this configuration. Thus, both the temperature and the relative humidity measurements were additionally corrected manually^105^. The time constants (*e*-folding time) applied for the temperature and the humidity sensor of the dropsonde type RD94 were 4 and 5 s, respectively. For the new dropsonde type RD41 used during MOSAiC-ACA, the time constants were characterized to be 1.3 and 1.6 s, respectively.

The humidity profiles show a dry-bias as they never reach a relative humidity of 100% inside clouds, which could be due to increasing contamination of the polymer film of the humidity sensor as the dropsondes age^106^. A reconditioning procedure aiming to correct for this bias has not been performed before each launch during the campaigns. The dropsonde data obtained during MOSAiC-ACA were thus corrected for the dry bias. An individual correction factor was applied to each humidity profile such that the saturation level of 100% is reached inside clouds. The correction factor is in the range of 1.025 for the majority of the sondes. This correction has not been done on the dropsonde data for AFLUX uploaded to PANGAEA.

The published datasets contain both the temperature and humidity data processed and corrected by ASPEN and the manually corrected data. However, data points above an altitude where the temperature sensor was not yet adjusted to the ambient temperature were removed from the datasets. The datasets are available in netcdf format and in a resolution of 1 Hz for nine flights of AFLUX^107^ and seven flights of MOSAiC-ACA^65^.

## Data Records

All datasets are published in PANGAEA with open access. Table 3 lists the corresponding dataset identifiers. Dataset collections of all corresponding datasets have been compiled, for both campaigns, AFLUX^26^ and MOSAiC-ACA^27^. With the exception of the nose boom and sun photometer data, that are available in compressed ascii format, all datasets have been converted to and are available in NetCDF4 file format. In general, each data file contains the data for one research flight. The files are identified by date and research flight number according to Table 1. An exception are the data of the fish-eye camera. With several gigabytes per hour, these data are very large and therefore provided in hourly files.

**Table 3:** Datasets, their identifiers on PANGAEA, and the data format.

Instrument	PANGAEA dataset ID		format
	AFLUX	MOSAiC-ACA	
Master tracks^19,20^	902876	924603	ascii
Nose boom^33,34^	945844	947787	ascii
Dropsondes^65,107^	921996	933581	netcdf
MiRAC-A^37,38^	944506	944507	netcdf
MiRAC-P^44^	944057	—	netcdf
HATPRO^45^	—	944101	netcdf
AMALi^48,49^	932455	932456	netcdf
SMART Albedometer^53^	—	933850	netcdf
AISA Eagle/Hawk^56,57^	930932	946965	netcdf
Fish-eye^61^	933839	933849	netcdf
Broadband & KT-19^66,67^	932020	936232	netcdf
Sun photometer^71^	946923	—	ascii
CAS/CDP, CIP, and PIP^80,81^	940564	940557	netcdf
2D-S & Polar Nephelometer^88,89^	941498	941538	netcdf
Nevzorov^81^	—	940557	netcdf

For full path append 10.1594/PANGAEA. (for example for the MiRAC-P data for AFLUX 10.1594/PANGAEA.944057). Collections of the datasets for AFLUX^26^ and MOSAiC-ACA^27^ are available on PANGAEA.

The datasets available on PANGAEA contain all necessary information needed to work with the data. If not provided within the respective dataset for the instruments, position and attitude can be extracted from the 100 Hz nose boom datasets^33,34^ and reduced to the resolution of the datasets (see Table 2).

## Technical Validation

The quality of the datasets has been assured by multiple steps. First, the instruments have been calibrated either before the installation into the aircraft in a laboratory, on ground during flight preparation before take-off, by specific flight patterns under well defined conditions during the research flights, or by cross-calibration with well calibrated instruments. Second, each instrument team conducted quality control by applying methods based on their respective user community standards. Most of the calibration procedures and the methods applied to ensure the quality of the collected data are described in the respective section, the data publication for the ACLOUD campaign^16^, in other peer-reviewed publications given in Table 2, or have been operated as supplied by the manufacturer.

**Fig. 5:**
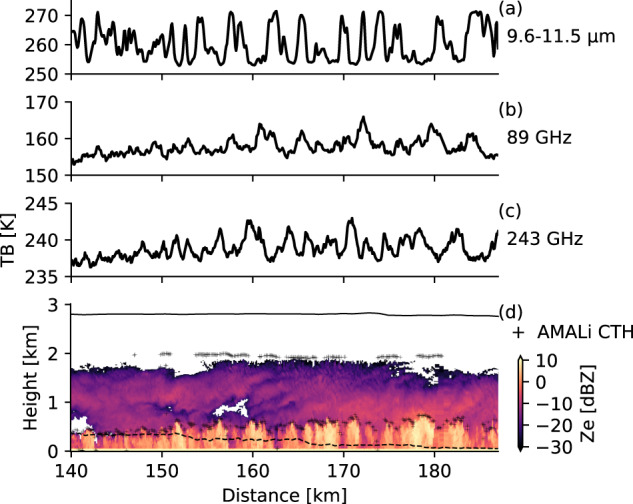
Remote sensing observations along the high-level segment shown in Fig. 4. Shown are the brightness temperatures measured by the (**a**) KT-19 infrared radiometer, and the (**b**) 89 and (**c**) 243 GHz from MiRAC-A and MiRAC-P, respectively, (**d**) radar reflectivities from MiRAC-A, cloud top altitudes from AMALi, and flight altitude (black and colored line). The flight altitudes of the high-level (solid black line) and in-situ shown in Fig. 6 (dashed black line) are indicated in (**d**).

**Fig. 6:**
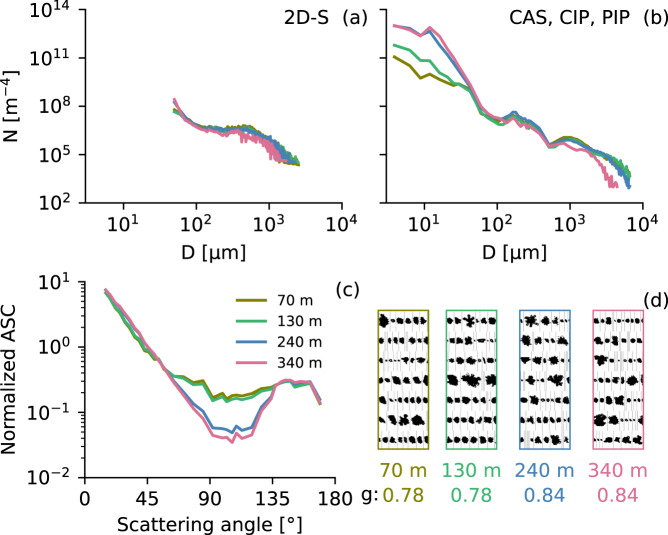
Cloud in-situ measurements along the segments shown in Fig. 4. Data presented for each of the four height levels (70 (olive), 130 (green), 240 (blue), and 340 m (pink)) include particle number size distributions from (**a**) 2D-S, and (**b**) combined CAS, CIP, and PIP probes, (**c**) normalized ASCs from Polar Nephelometer, and (**d**) a selection of images from 2D-S. The asymmetry parameter is indicated below the images in (**d**).

Figures 5, 6 illustrate the combination of the data collected by remote sensing and in-situ instruments operated on board Polar 5. The measurements are taken from two legs of RF09 along the flight path as shown in Fig. 4 carried out on the 01 April 2019 of the AFLUX campaign. To give an impression of the data collected, a flight section over open ocean was chosen were the aircraft was flying across roll clouds that are typical for marine cold air outbreaks. Since in-situ and remote sensing instrumentation is operated on the same platform, the measurements have to be performed one after the other. This resulted in a time difference of approximately 80 to 90 min between the two corresponding legs, i.e., in-situ (8:34 and 8:52 UTC) and remote sensing (10:02 and 10:12 UTC). Although, the separation in time is more than one hour, the in-cloud measurements can still be related well to the remote sensing observations. For example, the radar reflectivity in Fig. 5d shows the vertical structure of the cloud. Higher reflectivities are measured in the lower part of the clouds where the particles are larger as can be seen by the particle size distributions from the different in-situ probes shown in Fig. 6a,b. Measurements from lower parts of the cloud at 70 and 130 m altitude show more large particles and less smaller ones compared to the distributions collected in higher layers (240 and 340 m) where the radar reflectivity is lower. The normalized ASCs in Fig. 6c show a strong Mie forward peak and the images shown in d indicate that the higher reflectivities stem from snow particles. The cloud rolls can be nicely seen as well as areas of higher reflectivity in the lower 500 m. In the microwave radiometers Fig. 5b,c, these clouds are reflected by an increased brightness temperatures (higher emissivity of liquid in clouds than the one of the ocean surface), where as the KT-19 Fig. 5a shows a lower brightness temperature for the cloudy sections (clouds are colder than the surface).

**Fig. 7:**
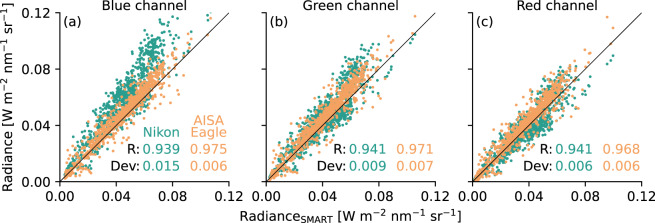
Comparison of radiances in nadir direction measured by SMART, Nikon, and AISA Eagle on 10 September 2020. *Dev* represents the root mean squared error between the reference radiance of SMART and the radiances by Nikon (green) and AISA Eagle (orange), respectively. *R* indicates the Pearson’s correlation coefficient.

To verify the individual calibrations and data quality, nadir radiances measured by SMART, AISA Eagle, and Nikon from RF09 during MOSAiC-ACA were compared. During a period of three hours, observations of cloud tops, with clouds, and above sea ice were performed, which cover a broad range of radiance values. Combining the three instruments needs to account for the different spatial, temporal, and spectral resolutions. AISA Eagle and SMART spectra were convoluted with respect to the spectral response functions of the three spectral channels of the Nikon camera. AISA Eagle and Nikon data were spatially averaged to match the size of the SMART footprint of 2°. Figure 7 displays scatterplots of the radiance data using SMART as a reference. The correlation between SMART and AISA Eagle data (orange dots) is consistent for all three channels with a correlation coefficient (R) of about 0.97 and an offset of 6%, which falls within the measurement uncertainty of the two instruments. The correlation coefficient between SMART and Nikon data is slightly lower with R = 0.94. The best agreement was found for the red and the green channels, while a significant offset of about 22% was derived for the blue channel. Finally, these findings were used to calibrate the Nikon camera in order to provide a consistent dataset.

## Usage Notes

During the field campaigns MOSAiC-ACA and AFLUX, a suite of remote sensing and in-situ instruments has been successfully operated on board the Polar 5 research aircraft to perform measurements of clouds, precipitation, and the structure of the lower Arctic atmosphere. The datasets collected can be used for a wide range of studies and are especially well suited for studies on Arctic mixed-phase clouds and boundary-layer processes, to derive higher level products by appropriate retrieval algorithms, or to perform model or satellite validation studies.

Along with the measurement campaigns conducted in the past years, the python package *ac3airborne*^23^ has been compiled to make the airborne data more visible and more readily usable. *ac3airborne* is a simple python module that follows the idea of the EUREC^4^A^108,109^ community. It is publicly available on github. The module makes use of the intake^110^ python library, that contains drivers for loading different file formats, cataloging system for specifying the sources of datasets as machine-readable YAML (YAML Ain’t Markup Language) files, and a server-client architecture to share the catalog meta data over the network. By that, all datasets of each instrument for every flight performed in (AC)^3^ are easily accessible without knowing their storage location or format. No additional information is needed. Everything else is handled by the package. Within the *ac3airborne* package, scripts are included that have been used to perform conversions on the publicly available datasets on PANGAEA for a better integration into the structure. A central part of the package is the flight segmentation^111^, where each research flight has been split up into logical parts like ascends, descends, specific patterns for in-situ probing, high, mid, or low level legs, and patterns for calibration purposes. By making use of this information defined by start and end time stamp of the specific section, it is easy to extract the data of interest.

The usage of the package together with a collection of example scripts is presented on *How to ac3airborne*^112^, an online and interactive jupyter book^113^. There, an overview of the data availability for each instrument on each flight is shown for all AC3 campaigns. The sections of the jupyter book describe simple usage cases of the datasets from reading procedures to quicklook production or more complex scripts for combining datasets from the different instruments. The use of the information provided by the flight segmentation is explained in more detail and its application shown along with the different code examples. For example, the data presented in Figs. 5 and 6 have been extracted and compiled using the opportunities given by *ac3airborne* for analyzing a flight with remote sensing and in-situ observations from the AFLUX campaign. The script is part of the example scripts in the online book.

## Data Availability

Each instrument is controlled either by code developed by the institution operating it or by code developed by the manufacturer and therefore often closed source or not even freely available and bundled with the instrument. Code used in the post-processing of the data has been developed by each institution, compiled in a package, and made available^114^. For the basic acquisition system of the Polar 5 aircraft and the KT-19, Werum Software & Systems AG has developed the software to communicate with the instruments and store the data. MiRAC-A radar, MiRAC-P, and HATPRO have been operated with software of the manufacturer Radiometer Physics GmbH. A LabView program by AWI controls AMALi and Nikon. The cloud particle probes CAS, CDP, CIP, and PIP are operated by a software from the manufacturer Droplet Measurement Technologies (DMT), where as for the 2DS it is Spec. Inc. and a LabView based program for the Polar Nephelometer. The spectral imager data acquisition software was developed by the manufacturer Specim, Spectral Imaging Ltd. Data evaluation was performed using the ENVI image analysis software. SMART is controlled by a LabView based software developed by Enviscope GmbH. The dropsonde system AVAPS has been post-processed with the Atmospheric Sounding Processing ENvironment (ASPEN, Version 3.4.4)^104^, which is publicly available. The ac3airborne package and tools developed within the project are written in python, open source, and publicly available on github^23^.
