# Author Correction: MOSAiC-ACA and AFLUX - Arctic airborne campaigns characterizing the exit area of MOSAiC

**DOI:** 10.1038/s41597-023-01980-z

**Published:** 2023-01-24

**Authors:** Mario Mech, André Ehrlich, Andreas Herber, Christof Lüpkes, Manfred Wendisch, Sebastian Becker, Yvonne Boose, Dmitry Chechin, Susanne Crewell, Régis Dupuy, Christophe Gourbeyre, Jörg Hartmann, Evelyn Jäkel, Olivier Jourdan, Leif-Leonard Kliesch, Marcus Klingebiel, Birte Solveig Kulla, Guillaume Mioche, Manuel Moser, Nils Risse, Elena Ruiz-Donoso, Michael Schäfer, Johannes Stapf, Christiane Voigt

**Affiliations:** 1 Institute for Geophysics and Meteorology, University of Cologne, Cologne, Germany; 2 Leipziger Institute for Meteorology, University of Leipzig, Leipzig, Germany; 3 Alfred-Wegener-Institut, Helmholtz-Zentrum für Polar- und Meeresforschung, Bremerhaven, Germany; 4 BreezoMeter, Haifa, Israel; 5 A.M. Obukhov Institute of Atmospheric Physics of the Russian Academy of Sciences, Moscow, Russia; 6 Laboratoire de Météorologie Physique, Université Clermont Auvergne/OPGC/CNRS, UMR 6016 Clermont-Ferrand, France; 7 Institute for Physics of the Atmosphere, Deutsches Zentrum für Luft- und Raumfahrt, Wessling, Germany; 8 Institute for Physics of the Atmosphere, Johannes Gutenberg University, Mainz, Germany

**Keywords:** Atmospheric science, Physics, Atmospheric science, Physics

Correction to: *Scientific Data* 10.1038/s41597-022-01900-7, published online 29 December 2022

In this article the funding from AWI was omitted. The original article has been corrected.

